# Comparative Efficacy of Platelet-Rich Fibrin and Enamel Matrix Derivative As Adjuncts to Non-Surgical Periodontal Therapy: A Split-Mouth Randomised Clinical Trial

**DOI:** 10.3290/j.ohpd.c_2750

**Published:** 2026-08-12

**Authors:** Nathan E. Estrin, Paras Ahmad, Savannah Laurey, Nima Farshidfar, Richard J. Miron

**Affiliations:** a Nathan E. Estrin The University of Iowa College of Dentistry and Dental Clinics, Iowa City, Iowa, USA; Department of Research, Advanced PRF Education, Jupiter, Florida, USA; Private Practice, Lakewood Ranch Dental, Sarasota, Florida, USA. Conceptualisation, methodology, formal analysis, investigation, resources, writing – original draft preparation, writing – review and editing, project administration, read and agreed to the published version of the manuscript.; b Paras Ahmad Department of Research, Advanced PRF Education, Jupiter, Florida, USA. Methodology, formal analysis, investigation, resources, writing – original draft preparation, writing – review and editing, read and agreed to the published version of the manuscript.; c Savannah Laurey Private Practice, Lakewood Ranch Dental, Sarasota, Florida, USA. Methodology, resources, writing – review and editing, read and agreed to the published version of the manuscript.; d Nima Farshidfar Department of Periodontology, University of Bern, Bern, Switzerland. Methodology, investigation, resources, writing – review and editing, read and agreed to the published version of the manuscript.; e Richard J. Miron Department of Research, Advanced PRF Education, Jupiter, Florida, USA; Private Practice, Lakewood Ranch Dental, Sarasota, Florida, USA; Department of Periodontology, University of Bern, Bern, Switzerland. Conceptualisation, methodology, formal analysis, investigation, resources, writing – original draft preparation, writing – review and editing, supervision, project administration, read and agreed to the published version of the manuscript.

**Keywords:** bone regeneration, dental enamel proteins, guided tissue regeneration, root planing, treatment outcome

## Abstract

**Background:**

Periodontitis is a chronic multifactorial inflammatory condition resulting in the gradual breakdown of periodontal supporting structures. Although non-surgical periodontal therapy (NSPT) is the gold standard first therapeutic option, its effectiveness can be limited in moderate-to-severe cases, hence necessitating the application of adjunctive therapies. Platelet-rich fibrin (PRF) and enamel matrix derivative (EMD) are among the most extensively utilised regenerative biomaterials; nevertheless, direct comparative evidence related to their adjunctive advantage in NSPT is scarce.

**Aim:**

This split-mouth randomised clinical trial compared the clinical effectiveness of EMD versus PRF as adjunct biological modalities to scaling and root planing (SRP) in the non-surgical management of Stage II/III and Grade A/B periodontitis.

**Methods and Materials:**

Fifteen participants meeting the eligibility criteria were included, with thirteen completing the trial. Each patient contributed two interproximal sites randomly allocated to either adjunctive EMD or PRF after full-mouth SRP. Before the placement of biomaterial, EMD sites were treated with ethylenediaminetetraacetic acid (EDTA), whereas PRF was prepared utilising the standard horizontal centrifugation approach. Periodontal probing depth (PD), gingival margin (GM) position, clinical attachment level (CAL), and bleeding on probing (BOP) were measured at baseline and 6-month follow-up.

**Results:**

Both biomaterials yielded significant intra-group improvements. Mean PD scores were reduced by 1.1 ± 0.6 mm in the EMD group and 1.2 ± 0.7 mm in the PRF group. CAL gains were 1.1 ± 0.7 mm and 1.1 ± 0.8 mm for EMD and PRF, respectively. GM levels remained stable across groups, and BOP markedly decreased (EMD: 77% to 23%; PRF: 85% to 31%). Intergroup comparisons revealed no statistically significant differences for PD (p > 0.999), GM (*P *= 0.174), or CAL (*P* = 0.556). Moreover, frequency distribution analysis showed that 100% of deep sites (≥ 6 mm) in the EMD group and 90% in the PRF group were reduced to < 6 mm at 6 months.

**Conclusion:**

Both EMD and PRF showed comparable clinical outcomes as adjunctive regenerative biomaterials in NSPT to manage moderate periodontal pockets. These outcomes support the versatility of clinicians in selecting either biological option based on practitioner and patient preference, availability, and cost, while opening avenues for future studies investigating their application in more complex periodontal cases.

Periodontitis is a chronic multifactorial disease characterised by a dysbiotic shift in the subgingival microbiome and an exacerbated inflammatory host response, eventually resulting in progressive destruction of the supporting periodontal tissues and, if left untreated, tooth loss.^3,37^ Beyond its local manifestations, periodontitis has been strongly linked with systemic comorbidities, such as adverse pregnancy outcomes, diabetes mellitus, and cardiovascular disease, further underscoring the significance of predictable and effective periodontal treatment.^1,2,10,23,42^

Non-surgical periodontal therapy (NSPT) remains the cornerstone of initial periodontal management, usually performed utilising ultrasonic scalers and hand instruments for disrupting and removing the subgingival biofilm and calculus deposits.^12,35^ While NSPT has proven efficacy in enhancing clinical attachment levels (CAL) and attenuating probing depth (PD), its limitations – especially in complex root morphologies, furcation areas, and deep pockets – have prompted the investigation of adjunctive approaches to optimise results.^25,41^ Over the past 20 years, a diverse array of adjunctive therapies has been introduced, including laser strategies, biologic mediators, and antimicrobial agents, each aiming to improve microbial control, host regulation, and wound healing.^4,15,22,38^

Among biologic agents, enamel matrix derivative (EMD), is one of the most widely investigated regenerative materials.^30,31^ Consisting mainly of enamel matrix proteins (EMPs), EMD has been used for around 30 years to enhance periodontal regeneration by activating periodontal ligament (PDL) fibroblast functionality, osteogenesis, and cementogenesis.^31^ Early pioneering reports by Lindskog et al^26^ exhibited EMPs’ role in cementum formation and root development. Considering its favourable safety profile and low immunogenicity, EMD has become a standard regenerative adjunct in both surgical and non-surgical approaches.^31^

Platelet concentrates, especially platelet-rich fibrin (PRF), represent another biologic adjunct having significant regenerative potential.^16,20,40^ First-generation platelet concentrates, including platelet-rich plasma (PRP), have a limitation of anticoagulant requirement, which interferes with the sustained release of growth factors and clot integrity.^7,27^ The introduction of PRF as a second-generation concentrate overcame this limitation by removing the anticoagulant and thus providing a solid fibrin matrix able to progressively release leukocytes, cytokines, and growth factors, hence supporting fibroblast proliferation, angiogenesis, and extracellular matrix remodelling.^17,19,39^ In the recent past, injectable PRF (i-PRF) has been introduced, allowing short-term liquid manipulation before clotting, hence improving biomolecule entrapment and supporting its application as a local drug delivery model.^18,28,29^

Despite the extensive clinical use of both PRF and EMD and their increasingly recognised roles as biologic adjuncts in NSPT, direct comparative evidence between these two agents remains scarce. Present investigations primarily assess each biomaterial independently, leaving a vital gap in comprehending their comparative effectiveness. Hence, the current split-mouth randomised clinical trial (RCT) aims to directly compare the adjunctive application of PRF and EMD in the non-surgical management of Stage II/III, Grade A/B periodontitis.

## METHODS AND MATERIALS

### Ethical Considerations and Study Design

The study protocol was submitted, reviewed, and approved by the Sterling Institutional Review Board (IRB approval ID: 12625-NEstrin). This split-mouth RCT was conducted in a private practice setting (Lakewood Ranch Dental, Florida, USA). All procedures were conducted following the Declaration of Helsinki (2013 revision).^6^ Before the enrollment of study participants, written informed consent was obtained.

### Study Participants and Eligibility Criteria

A total of 15 patients were recruited between December 2024 and April 2025. During the initial screening visit, each participant underwent a comprehensive assessment that included a detailed medical and dental history, full-mouth periodontal charting, and a complete series of bitewing and peri-apical radiographs to confirm eligibility.

Inclusion criteria comprised: (i) adults aged 18–65 years; (ii) diagnosis of Stage II/III periodontitis, Grade A/B progression with at least six interproximal sites showing PD > 4 mm (excluding third molars) according to the 2017 World Workshop classification^44^; and (iii) presence of adequate interproximal sites to allow allocation to both treatment groups within the split-mouth design.

Exclusion criteria comprised: (i) previous periodontal treatment within the past 6 months; (ii) local or systemic antibiotic treatment within the past 3 months; (iii) diagnosis of Stage I/IV Periodontitis; (iv) poor oral hygiene defined as a full-mouth plaque score > 30%; (v) history of radiation or immunosuppressive therapy; (vi) presence of neurologic disorders, major mechanical obstruction to mouth opening, or acute temporomandibular capsulitis; (vii) systemic conditions that may compromise bone metabolism or wound healing such as metabolic bone disease, uncontrolled diabetes; (viii) history of substance abuse such as drug or alcohol; or (ix) current pregnancy, nursing, or planning pregnancy during the study period.

### Randomisation, Study Groups, and Baseline Clinical Evaluation

A split-mouth design was used in which individual patients contributed sites to all study groups. Random allocation of interproximal sites ensured that individual patients acted as their own control, minimising inter-individual heterogeneity. Two sites were randomly assigned to the adjunctive therapy groups. The following two treatment modalities were assessed: (i) SRP using ultrasonic instruments and hand curettes with adjunctive EMD application; and (ii) SRP with adjunctive PRF application. At the baseline visit, periodontal parameters were measured, including PD, gingival recession (GR), and CAL.

### Intervention Protocols

#### Scaling and root planning

After administering local anaesthesia, all patients underwent full-mouth SRP utilising ultrasonic instrumentation, hand curettes, and adjunctive laser therapy (Fotona, Ljubljana, Slovenia), which was specifically utilised to facilitate the eradication of residual granulation tissue. Root surfaces were thoroughly debrided until smoothness was confirmed with a periodontal explorer.

#### EMD application

For the EMD group, one randomly chosen site received application of EMD (Emdogain®, Institut Straumann, Basel, Switzerland) delivered via a 0.3 ml pre-filled syringe until visible pocket overflow was observed. Before EMD placement, the site was treated with ethylenediaminetetraacetate (EDTA; Institut Straumann, Basel, Switzerland) to eradicate the smear layer, followed by copious saline irrigation.

#### PRF preparation and application

PRF was prepared following the manufacturer’s instructions (Bio-PRF, Florida, USA). Two 10 ml solid-PRF tubes (red) and two liquid-PRF tubes (blue) were collected by venipuncture utilising a 21G butterfly needle and centrifuged concurrently for 8 min at 700 × g employing a horizontal centrifuge system (Bio-PRF, Florida, USA). After collecting the PRF layer, it was transferred to a sterile metal dish and allowed to polymerise at room temperature for 10 to 20 min. After clotting, 2 to 3 small fragments of PRF were incorporated into the designated sites subgingivally utilising a cord packer.

### Postoperative Care, Follow-Up, and Outcome Assessment

Postoperatively, patients were scheduled for a 2-week follow-up for reinforcement of oral hygiene instruction. A supportive periodontal maintenance appointment was conducted at 3 months. At the 6-month follow-up, periodontal parameters, such as PD, GR, and CAL, were re-evaluated and compared with baseline measurements to assess treatment outcomes.

### Sample Size Calculation

The sample size for this trial was determined using data from the study by Sybil et al^43^ as a reference. Calculations were performed using the online platform Sealed Envelope (https://www.sealedenvelope.com/power/continuous-superiority/). In the referenced study, the standard deviation (SD) for the control group was reported as 0.55 (n = 25). To account for methodological variations and ensure a conservative estimate, an evidence value (d) of 1 was opted. Based on a significance level of 5% (α = 0.05) and a statistical power of 80% (1-β = 0.8), the minimum sample size necessary was calculated as 13 participants per group. Permitting for an anticipated dropout rate of 20%, the final target allocation was set at 15 participants, with individual patients contributing sites for both test groups (PRF and EMD) under the split-mouth design.

### Statistical Analysis

Data were analysed using GraphPad Prism version 10.0 (GraphPad Software, San Diego, CA, USA). Continuous variables, such as PD, GM, and CAL, were expressed as mean ± SD, while categorical variables (BOP) were expressed as frequencies and percentages. The Shapiro–Wilk test was used to assess the normality. Suitable for the split-mouth design of the present study, a paired t-test was used to assess the inter-group comparisons (EMD versus PRF). A P value of < 0.05 was considered statistically significant.

## RESULTS

### Primary Characteristics of Study Participants

A total of 15 participants (9 males and 6 females) were initially enrolled in the study. The mean age of the study participants was 46.7 ± 10.8 years, ranging between 30 and 63 years. Six participants (40%) were current smokers, whereas the remaining 9 (60%) were non-smokers. Each patient contributed two interproximal sites for the trial; one was randomly allocated to adjunctive EMD and the other to the adjunctive PRF. The chosen sites were distributed across the maxillary and mandibular arches, including premolars and molars. During the trial, one participant was lost to follow-up, and another was excluded owing to a heavy plaque score (100%) at the 3-month periodontal maintenance visit. Eventually, 13 participants were included in the final analysis (Table 1).

**Table 1:** Primary features of the study participants

Serial no.	Gender	Age (Years)	Smoking status
1	Male	55	Yes
2	Female	62	Yes
3	Male	63	Yes
4	Male	37	No
5	Female	56	Yes
6	Female	43	Yes
7	Male	40	No
8	Male	36	No
9	Male	30	No
10	Female	46	No
11	Male	32	No
12	Male	48	No
13	Male	57	No


### Periodontal Clinical Parameter Outcomes

#### Probing depth outcomes

**Fig 1a to f:**
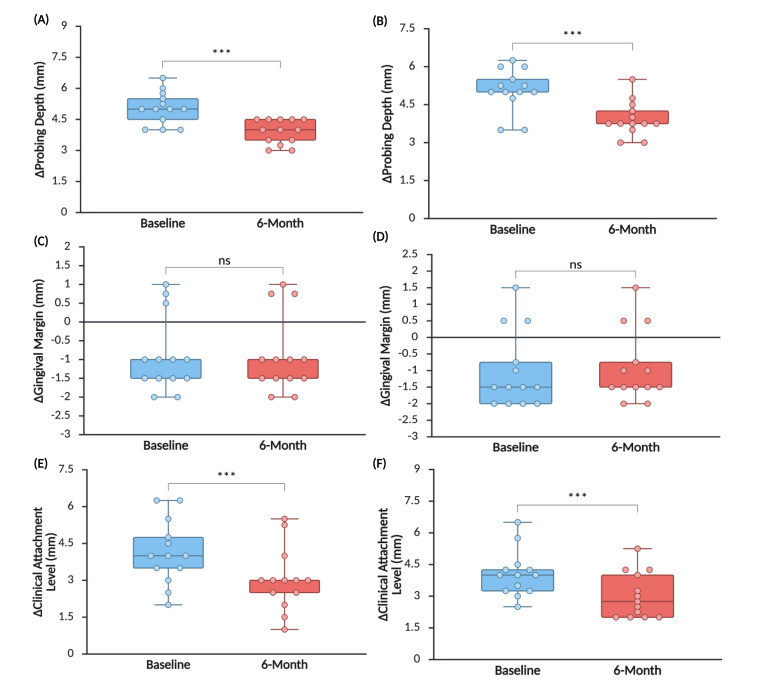
Probing depth scores at baseline and 6-month follow-up for (a) adjunct EMD; and (b) adjunct PRF groups. Gingival margin scores at baseline and 6-month follow-up for (c) adjunct EMD; and (d) adjunct PRF groups. Clinical attachment level scores at baseline and 6-month follow-up for (e) adjunct EMD; and (f) adjunct PRF groups.

**Fig 2a to c:**
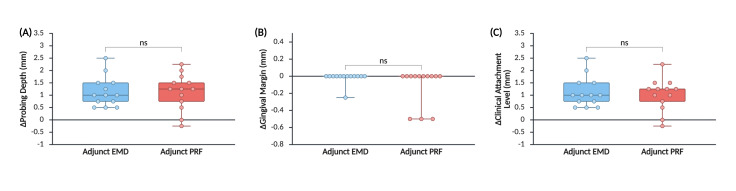

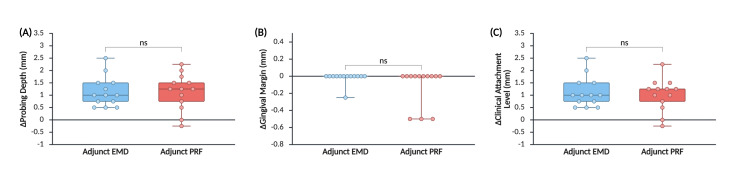
Changes in (a) probing depth; (b) gingival margin; and (c) clinical attachment level scores between adjunct EMD and adjunct PRF groups.

Intra- and inter-group analyses showed that both EMD and PRF provided comparable clinical improvements over the 6-month follow-up. PD was significantly reduced within each group: from 5.04 ± 0.78 mm to 3.90 ± 0.59 mm in the EMD group (Fig 1a), and from 5.08 ± 0.84 mm to 3.94 ± 0.69 mm in the PRF group (Fig 1b), demonstrating a similar mean decrease of 1.13 ± 0.2 mm (EMD: *P* < 0.0001; PRF: *P* < 0.001). Intra-group comparison showed no statistically significant difference in PD reduction at 6 months (*P* > 0.99; Fig 2a).

#### Gingival margin outcomes

GM remained largely stable across both research groups, with no statistically significant intragroup changes (EMD: *P* = 0.337 [Fig 1c]; PRF: *P* = 0.082 [Fig 1d]) and no significant inter-group differences at 6 months (*P* = 0.174; Fig 2b).

#### Clinical attachment level outcomes

Both groups demonstrated significant CAL gains. CAL improved from 4.13 ± 1.31 mm to 3.02 ± 1.29 mm in the EMD group (Fig 1e) and from 4.06 ± 1.09 mm to 3.04 ± 1.08 mm in the PRF group (Fig 1f), corresponding to gains of 1.12 ± 0.61 mm (EMD; *P* < 0.0001) and 1.02 ± 0.68 mm (PRF; *P* < 0.001). Inter-group comparison demonstrated no statistically significant difference at 6 months (*P* = 0.556; Fig 2c).

#### Bleeding on probing outcomes

BOP improved markedly in both groups. In the EMD group, the proportion of BOP-positive sites decreased from 77% at baseline to 23% at 6 months, while in the PRF group, the proportion decreased from 85% to 31% (data not shown), suggesting a substantial decrease in gingival inflammation.

#### Frequency distribution of deep periodontal pockets (≥ 6 mm)

At baseline, the number of sites presenting with PD ≥ 6 mm was 17 in the EMD group and 20 in the PRF group. At the 6-month follow-up, no sites in the EMD group and 2 sites in the PRF group remained ≥ 6 mm. Importantly, all 17 sites (100%) in the EMD group and 18 out of 20 sites (90%) in the PRF group improved from ≥ 6 mm to < 6 mm. Only 2 sites (10%) in the PRF group remained ≥ 6 mm at 6 months.

## DISCUSSION

The present split-mouth RCT assessed, for the first time, the direct comparison of EMD and PRF as adjunct biologics in NSPT for Stage II/III, Grade A/B periodontitis. Both biologics showed significant enhancements in PD, GM stability, CAL, and BOP at 6 months. Importantly, no statistically significant differences were found between EMD and PRF at any time point, reflecting that both biomaterials provided comparable clinical outcomes when combined with SRP.

These results are in accordance with the increasing body of literature exhibiting the effectiveness of bioactive adjuncts in improving periodontal healing after NSPT.^48^ Notwithstanding their unique biological mechanisms, EMD and PRF seemingly resulted in comparable clinical endpoints in moderately deep periodontal pockets. EMD has been widely acknowledged as one of the most thoroughly investigated periodontal regenerative materials, with an established capability to enhance early wound stabilisation, PDL cell proliferation, and cementogenesis via biomimicry of natural tooth development.^11,13^ Its success, especially in intrabony defects via surgical approach, has established a rigorous benchmark for biologic adjuncts.^11,32^ On the contrary, PRF acts through an autologous fibrin scaffold enriched with cytokines, leukocytes, and growth factors that are released progressively over 10 to 14 days.^33,38^ This prolonged release plays a vital part in promoting fibroblast activity, enhancing angiogenesis, and regulating inflammation.^24,46^

In the current report, the similarity in results between the two treatment modalities is noteworthy considering their fundamentally divergent modes of action. One plausible explanation is the moderate severity of the involved sites. The baseline PD averaged around 5 mm for both groups, situated within the range where SRP alone is reported to provide reliable clinical enhancements. As demonstrated by Cobb et al,^9^ deeper pockets (> 7 mm) show significantly higher reductions after intervention, while moderate defects exhibit more limited change. This restricted “ceiling effect” may have alleviated the possibility of identifying variations between adjuncts.^9^ Hence, the full regenerative potential of either EMD or PRF may not be completely manifested in moderate-depth pockets.

Beyond the severity of the disease, it is important to recognise numerous methodological considerations. EDTA root conditioning was conducted in the EMD group, as mandatory for optimal protein adsorption; however, not in the PRF cohort. Independent investigations have reported that EDTA effectively exposes collagen fibres, improves smear layer eradication, and enhances root surface biocompatibility.^5,21,47^ This might inadvertently confer a benefit to the EMD group. Prospective studies should contemplate the standardisation of EDTA application across both cohorts to isolate the impacts of the biomaterial itself. Even though EMD is conventionally most efficacious under dry conditions with flap access,^45^ its flapless application in this study indicates an evolving trend toward minimally invasive regenerative treatment. Prior systematic reviews report that flapless EMD provides results in smaller gains in comparison with surgical access, potentially owing to diminished visualisation and moisture control.^8,14^ Nevertheless, this study exhibits that EMD can perform comparably to PRF even under such limitations, suggesting that biological advantages may still be achieved using minimally invasive approaches.

Clinically, the present findings suggest that both EMD and PRF may be utilised as adjunctive biologic options during NSPT. Accordingly, treatment selection may be based on clinician preference, patient-related factors, cost, availability, and handling characteristics. The effectiveness of EMD is influenced by its adsorption to the conditioned root surface, and for this reason it is ideally applied under relatively dry conditions, which may be more technique-sensitive during surgical application. In contrast, PRF does not rely on root condition/adsorption and rather provides a fibrin-rich scaffold for gradual release of growth factors to support wound healing. Therefore, PRF may offer a practical advantage when moisture control is difficult. Nevertheless, both materials should be considered adjuncts to meticulous debridement rather than substitutes.

The significant decrease in BOP found in both groups highlights the anti-inflammatory effects of adjunct biologics. The leukocyte composition of PRF probably contributes to its regulatory impacts on the expression of inflammatory cytokines,^36^ while EMD has been demonstrated to alleviate pro-inflammatory cytokines, including tumour necrosis factor-alpha (TNF-α) and interleukin 1-beta (IL-1β).^34^ Clinically, this decrease in soft-tissue inflammation reflects a vital factor of periodontal stability and long-term prognosis.

Moreover, this study benefits from a split-mouth methodology, which inherently minimises interindividual variability. However, its drawbacks must be recognised. The limited sample size, though sufficiently powered for the anticipated effect size, limits generalisability. The follow-up, limited to 6 months, may not capture the complete regenerative potential of either biomaterial. Furthermore, the inclusion of smokers may have diminished therapeutic effects despite balanced allocation.

Within these limitations, the outcomes indicate that both EMD and PRF are comparably efficacious adjuncts to NSPT in moderate periodontitis. Their biological distinctions may become more pronounced in more intricate or deeper defects, necessitating future research involving longer follow-ups, furcation involvements, and intrabony defects. Moreover, mechanistic investigations assessing root-biomaterial interplay in surgical versus flapless conditions would elucidate clinical indications for each biomaterial.

## CONCLUSION

Both EMD and PRF showed comparable clinical outcomes as adjunctive regenerative biomaterials in NSPT to manage moderate periodontal pockets. These outcomes support the versatility of clinicians in selecting either biological option based on practitioner experience, availability, cost factors, and patient preference, while opening avenues for future studies investigating their application in more complex periodontal challenges.

### Acknowledgements

Nima Farshidfar is a recipient of the 2024 Research Scholarship from Osteology Foundation.

#### Conflict of interest

Richard J. Miron holds intellectual property on the production of PRF and is the founder of Miron Research and Development in Dentistry LLC. All other authors declare that they have no conflict of interest.

#### Data availability statement

Data available on request from the authors

#### Funding

This research was not supported by any funding.
